# Crisscross Flower Fertilization Optimization (CCFFO): A Bio-Inspired Metaheuristic for Global and Reservoir Production Optimization

**DOI:** 10.3390/biomimetics10090633

**Published:** 2025-09-19

**Authors:** Xu Wang, Jingfu Shan

**Affiliations:** 1 School of Geosciences, Yangtze University, Wuhan 430100, China; 2022730034@yangtzeu.edu.cn; 2 Key Laboratory of Exploration Technologies for Oil and Gas Resources, Yangtze University, Wuhan 430100, China

**Keywords:** flower fertilization optimization, metaheuristics, crisscross strategy, reservoir production optimization, global optimization

## Abstract

Developing solutions for complex optimization problems is fundamental to progress in many scientific and engineering disciplines. The Flower Fertilization Optimization (FFO) algorithm, a powerful metaheuristic inspired by the reproductive processes of flowering plants, is one such method. Nevertheless, FFO’s effectiveness can be hampered by a decline in population diversity during the search process, which increases the risk of the algorithm stagnating in local optima. To address this shortcoming, this work proposes an improved method called Crisscross Flower Fertilization Optimization (CCFFO). It enhances the FFO framework by incorporating a crisscross (CC) operator, a mechanism that facilitates a structured exchange of information between different solutions. By doing so, CCFFO effectively boosts population diversity and improves its capacity to avoid local optima. Rigorous testing on the challenging CEC2017 benchmark suite confirms CCFFO’s superiority; it achieved the top overall rank when compared against ten state-of-the-art algorithms. Furthermore, its practical effectiveness is demonstrated on a complex reservoir production optimization problem, where CCFFO secured a higher Net Present Value (NPV) than its competitors. These results highlight CCFFO’s potential as a powerful and versatile tool for solving complex, real-world optimization tasks.

## 1. Introduction

Effective decision-making is the bedrock of success in any complex system, whether in technological design, financial management, or logistical planning [1]. In a quantitative framework, this translates to the search for an optimal solution within a constrained, high-dimensional decision space—a process formally known as optimization. The goal is to systematically identify a course of action that yields the best possible outcome according to a predefined metric of success [2]. From scheduling airline routes to minimize fuel consumption to designing drug molecules for maximum therapeutic effect, the impact of superior optimization is profound. As the scale and complexity of these problems escalate, they invariably push beyond the limits of human intuition and conventional analytical methods, creating a critical and ever-growing demand for advanced computational tools that can navigate these intricate challenges effectively [3,4,5].

Mathematically, these real-world challenges manifest as objective functions of formidable complexity. They are frequently non-differentiable, discontinuous, and defined over a high-dimensional domain where the number of potential solutions grows exponentially with the number of variables [6]. The solution landscape is typically multimodal, populated by a vast number of local optima that can mislead an optimizer away from the true global best [7]. The presence of intricate, often nonlinear constraints further complicates the search by creating disjoint feasible regions and making it difficult to even identify a valid solution, let alone the optimal one. This inherent structural complexity forms a significant barrier, rendering many classical optimization techniques either inapplicable or inefficient [8,9,10].

To navigate such challenges, a well-established portfolio of classical optimization techniques has been historically employed, encompassing gradient-based approaches like the conjugate gradient method [11] and direct search algorithms such as the simplex method [6]. The power of these methods stems from their rigorous mathematical foundations, which often guarantee convergence under specific, idealized conditions [1]. However, this very reliance on idealized properties becomes their critical vulnerability. When confronted with the non-convex, multimodal, and often discontinuous nature of real-world problems, their theoretical guarantees evaporate. Gradient-based methods, being inherently local in scope, frequently get trapped in the nearest suboptimal peak, while direct search and programming methods often become computationally intractable as problem dimensionality increases—a phenomenon famously known as the “curse of dimensionality” [12].

To address the shortcomings of classical methods, researchers have increasingly turned to a powerful family of solution techniques: metaheuristic algorithms [13,14]. These algorithms were conceived specifically to tackle the kind of complex optimization problems that are intractable for traditional approaches. Free from the restrictive requirements of continuity or differentiability, metaheuristics employ sophisticated stochastic operators to explore the solution space globally [15]. This means they interact with a problem solely by providing a potential solution and receiving a corresponding fitness value, making them directly applicable to a wide range of problem representations, including complex simulations or surrogate models. They achieve a robust search performance by mimicking successful strategies found in nature, such as Darwinian evolution or the foraging behavior of swarms. This nature-inspired foundation grants them the remarkable ability to avoid premature convergence and effectively hone in on high-quality solutions in even the most challenging optimization landscapes [16].

The diverse strategies employed by metaheuristics generally fall into two major streams of thought. The first is rooted in evolutionary theory, called Evolutionary Algorithms (EAs) [7]. These algorithms, such as the widely used Genetic Algorithm (GA) [17] and Differential Evolution (DE) [18], iteratively breed superior solutions by applying operators that mimic genetic selection, crossover, and mutation. The second stream is inspired by the emergent problem-solving capabilities of social colonies, forming the basis of Swarm Intelligence (SI). Prominent examples like Particle Swarm Optimization (PSO) [19] and Ant Colony Optimization (ACO) [20] leverage a population of interacting agents that follow simple local rules, which collectively guide the entire swarm towards the global optimum.

Despite the proliferation and demonstrated success of these diverse metaheuristics, the No Free Lunch (NFL) theorem [21] mathematically demonstrates that, when averaged across all possible optimization problems, no single algorithm can outperform any other. This fundamental principle indicates that there is no universally superior or “one-size-fits-all” optimizer. An algorithm’s strong performance on one type of problem often comes at the expense of its effectiveness on another. This insight highlights the importance of continuing to explore and design novel or improved algorithms specifically adapted to address particular complex challenges more efficiently.

The principles of the NFL theorem are particularly evident in reservoir production optimization [22], a domain where the need for tailored algorithms is paramount. The primary objective in this high-stakes field is to maximize the economic value of an oilfield, typically quantified by the Net Present Value (NPV). This is achieved by strategically managing injection and production wells throughout the reservoir’s lifetime [23]. This task is exceptionally complex due to profound subsurface heterogeneity, highly nonlinear multiphase fluid dynamics, and a vast, high-dimensional decision space. Consequently, classical optimization methods that depend on simplified assumptions or local gradient information are fundamentally ill-equipped to handle the rugged, computationally expensive, and constrained nature of this problem, thereby creating a fertile ground for the application of advanced metaheuristic approaches.

Scholarly efforts in reservoir production optimization have largely bifurcated into two primary thrusts: developing high-level strategies to improve overall optimization efficiency and direct enhancement of the core optimization algorithms themselves. The first and arguably most critical research direction addresses the immense computational burden of full-physics simulations, which is the primary bottleneck in practical applications. The most prominent strategy in this category is the use of surrogate-assisted frameworks. For instance, Wang et al. [24] introduced a multi-surrogate framework (MSFASM) that adaptively selects models to reduce reliance on expensive evaluations. Beyond simply replacing simulations, another powerful efficiency-driven approach is knowledge transfer, where insights from previously solved problems are leveraged to accelerate new optimizations. The competitive knowledge transfer (CKT) method by Cao et al. (2023) [25] exemplifies this by intelligently reusing historical development schemes. The scope of this efficiency-focused research also extends to redefining the problem and solution paradigm. Oliver et al. (2025) [26], for example, tackled the multi-objective problem of balancing economic profit and CO_2_ emissions, while Zahedi-Seresht et al. (2024) [27] introduced a novel Q-learning approach from reinforcement learning to find optimal production rates in a model-free manner. These methods collectively aim to make the optimization process more tractable and cost-effective. While the aforementioned strategies enhance efficiency, another vital research thrust focuses on fundamentally improving the internal mechanics of the core optimization algorithms to bolster their search capabilities. A notable trend here is the integration of hybrid strategies to achieve a better balance between exploration and exploitation. These efforts seek to make the algorithms inherently more powerful, enabling them to find better solutions within a given computational budget.

Among the array of nature-inspired algorithms, the Flower Fertilization Optimization (FFO) algorithm [28], which simulates the pollination process of flowering plants, has distinguished itself as a particularly effective method. Its core strength lies in a dual-search mechanism that elegantly models both global pollination (cross-pollination) for exploration and local pollination (self-pollination) for exploitation. This bio-inspired design allows FFO to maintain a robust balance, leading to its successful application across numerous optimization benchmarks. However, a closer examination of its search operators reveals a structural limitation. The primary update mechanism guides individuals largely based on their own trajectory and the single global best solution, thereby offering limited channels for direct, peer-to-peer information exchange within the population. This relative isolation of search agents can lead to a gradual decline in population diversity as the search progresses, increasing the risk of premature convergence, especially on complex, deceptive landscapes. Therefore, enhancing inter-solution communication within the FFO framework presents a clear and promising avenue for improvement.

The motivation for this work stems from a structural weakness in the standard Flower Fertilization Optimization (FFO) algorithm. While FFO effectively balances global and local search, it has a significant limitation: individuals in the population primarily learn from the single global best solution. This approach restricts direct information sharing between other solutions. As a result, the population can lose diversity over time, making the algorithm prone to premature convergence, where it gets stuck on a suboptimal solution. To address this limitation, we propose the Crisscross Flower Fertilization Optimization (CCFFO). Our method introduces a crisscross (CC) operator that enables direct and structured information exchange between different individuals. We hypothesize that by improving inter-solution communication, CCFFO can better maintain population diversity, avoid premature convergence, and achieve superior optimization performance.

The contributions of this work are as follows:

- A novel Crisscross Flower Fertilization Optimization (CCFFO) algorithm is introduced. By combining the balanced search strategy of FFO with a crisscross operator, CCFFO enhances population diversity, accelerates convergence, and improves overall optimization effectiveness.
- The proposed method is thoroughly evaluated on the CEC2017 benchmark suite, where it is compared against a wide range of established metaheuristics. Its superiority is further confirmed through rigorous statistical analyses, including the Friedman and Wilcoxon signed-rank tests.
- The practical value of CCFFO is demonstrated in a reservoir production optimization case study. Results show that the algorithm achieves higher economic returns (NPV) and exhibits strong robustness in tackling real-world optimization problems.

The remainder of this paper is organized as follows: Section 2 reviews the original FFO algorithm. Section 3 introduces the proposed CCFFO method and the integration of the crisscross operator. Section 4 presents the experimental design, benchmark results, and statistical evaluations. Section 5 applies CCFFO to reservoir production optimization. Finally, Section 6 concludes the work with a summary and directions for future research.

## 2. The Original FFO

The FFO algorithm, a metaheuristic algorithm proposed in 2025 by Albedran et al. [28], was inspired by the natural fertilization processes of flowering plants. The FFO algorithm emulates the journey of pollen grains towards ovules by integrating several key mechanisms: a global search strategy powered by Lévy flights to model long-distance pollination, a local search phase characterized by velocity reduction to simulate final approach, and a population mixing and survival strategy based on elitism. The primary mathematical model of the FFO algorithm is structured as follows:

**1. Global Search via Lévy Flights:** The global exploration capability of FFO is inspired by the long-distance dispersal of pollen grains. This natural process, where pollen is carried by wind or pollinators, allows for the exploration of new territories far from the origin. Specifically, the random walk pattern of Lévy flights, which is characterized by a series of short steps combined with occasional long-distance jumps, effectively mimics how a pollinator like a bee might travel locally before making a long flight to a new patch of flowers, or how a gust of wind can carry pollen unpredictably far. This ability to make large, abrupt moves across the search space is what underpins the algorithm’s global exploration capability. FFO models this using Lévy flights, a random walk pattern that enables large jumps across the search space, effectively preventing the algorithm from becoming trapped in local optima. The global search component influences the update of a solution’s position *X* using a step size *L* derived from a Lévy distribution.

(1) $$L=\frac{u}{|v{|}^{1/\xi }}$$

where u ∼ N(0, σ2) and v ∼ N(0, 1), σ is calculated as

(2) $$\sigma ={\left(\frac{\Gamma (1+\xi )\cdot sin(\pi \xi /2)}{\Gamma (\frac{1+\xi }{2})\xi \cdot 2^{\frac{\xi -1}{2}}}\right)}^{1/\xi }$$

where Γ is the Gamma function and ξ is the Lévy distribution parameter, which is typically set in the interval, with 1.5 being a common value.

**2. Local Search and Guided Movement:** FFO’s local exploitation mechanism mimics the final, focused stage of fertilization. As a pollen grain nears an ovule, its movement slows and becomes more directed. The algorithm models this by reducing the velocity of solutions and guiding them towards the best-performing regions of the search space. This is achieved through two complementary actions: a velocity reduction controlled by a damping factor and a movement vector that pulls solutions towards a collective center defined by the best (*X_first*), worst (*X_end*), and median (*X_middle*) solutions in the current population. The velocity *V* is updated as:

(3) $$V_{i}^{t+1}=V_{i}^{t}e^{\left(\frac{-1}{\gamma^{t+1}}\right)}$$

where the reduction coefficient *γ* is damped over iterations *t* by a factor *β*. The overall position update integrates this local search with the global exploration of Lévy flights into a unified rule, as detailed in the original literature [28].

**3. Population Mixing and Survival Strategy:** To ensure the propagation of high-quality solutions and maintain a healthy level of competition, FFO employs a population mixing and survival strategy inspired by the concept of elitism. In nature, the fittest pollen grains are most likely to succeed. FFO simulates this by combining the newly generated solutions (the newpollen population) from the current iteration with the existing parent population. This merged group, containing both old and new individuals, is then subjected to a competitive exclusion process. The individuals are sorted based on their fitness (cost value), and only the top-ranking solutions—equivalent to the original population size—are selected to survive and proceed to the next generation. This mechanism guarantees that the best solution found at any point in the search is never lost and provides a strong selective pressure that continually guides the population towards more optimal regions of the search space.

**Figure 1:**
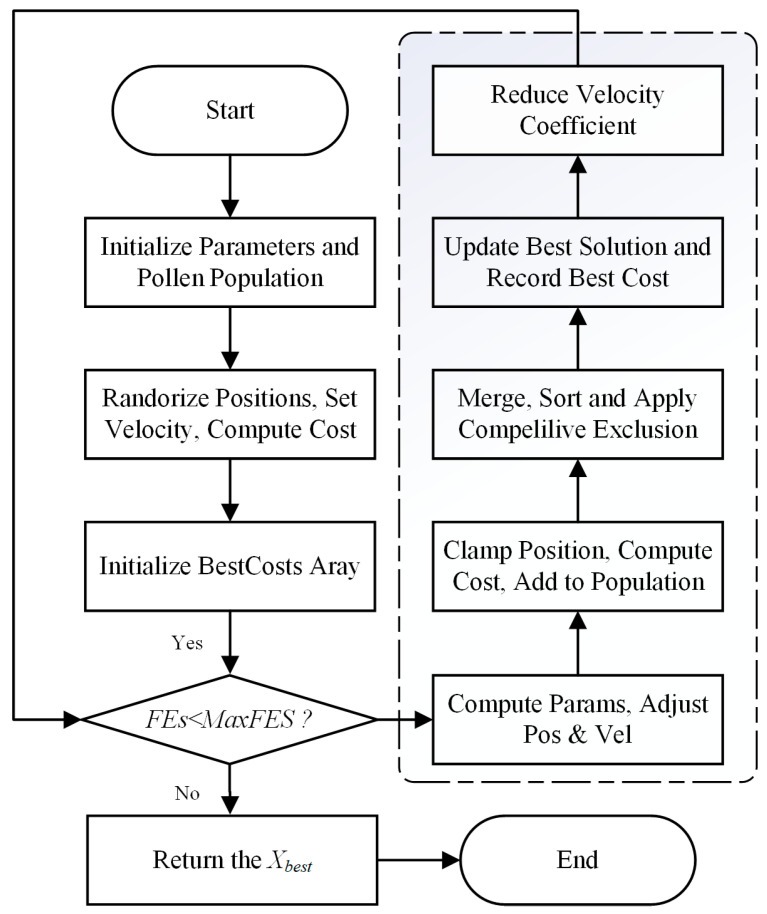
Flowchart of the FFO.

The flowchart of FFO is shown in Figure 1.

In the figures, “FES” denotes the current number of function evaluations, while “MaxFES” represents the maximum number of function evaluations allowed.

## 3. Proposed CCFFO

### 3.1. Crisscross Strategy

The CC strategy, first conceptualized within the Crisscross Optimization (CSO) algorithm, is a sophisticated search mechanism designed to enhance population diversity and accelerate convergence. It achieves this by implementing two distinct yet complementary search modalities: Horizontal Crossover (HC) and Vertical Crossover (VC). The HC modality facilitates a rich exchange of information between different individuals, enabling the population to collaboratively explore the search space. Conversely, the VC modality promotes exploration within a single individual by creating novel solutions from its own dimensional components. By integrating this dual-axis crossover strategy into the FFO framework, we aim to overcome the limitations of its original update rule, providing a more structured and potent mechanism for generating trial solutions and effectively avoiding premature stagnation in local optima.

(4) $$O_{a,d}=\rho_{1}\cdot P_{a,d}+(1-\rho_{1})\cdot P_{b,d}+\alpha \cdot (P_{a,d}-P_{b,d})$$

where $\rho_{1}$ is a uniformly distributed random number in [0, 1], and α is a random scaling factor in [−1, 1] that controls the contribution of the difference vector. The scaling factor a allows for both interpolation and extrapolation, enhancing the algorithm’s exploratory power. However, this process can generate new solutions that fall outside the predefined search boundaries. To ensure all solutions remain within the feasible region, a clamping mechanism is applied. If any dimension of a newly generated offspring exceeds the search limits, its value is reset to the nearest boundary. A similar operation is performed to generate a second offspring from $P_{b}$.

Subsequently, the Vertical Crossover is applied. For a given parent solution $P_{a}$ two distinct dimensions, $d_{1}$ and $d_{2}$, are chosen at random. The algorithm generates a new offspring by interpolating between them:

(5) $$O_{a,d1}^{\prime }=\rho_{2}\cdot P_{a,d1}+(1-\rho_{2})\cdot P_{a,d2}$$

where $\rho_{2}$ is another random number in [0, 1]. This new value replaces the original $P_{a,d1}$ creating a new candidate solution. Following crossover in both HC and VC, a greedy selection mechanism is applied. This strategy ensures that for each parent-offspring pair, only the solution with superior fitness survives into the subsequent generation.

### 3.2. The Proposed CCFFO

This section introduces the proposed Crisscross Flower Fertilization Optimization (CCFFO) algorithm. The CCFFO framework enhances the standard FFO by strategically integrating a crisscross (CC) operator into its iterative loop.

**Figure 2:**
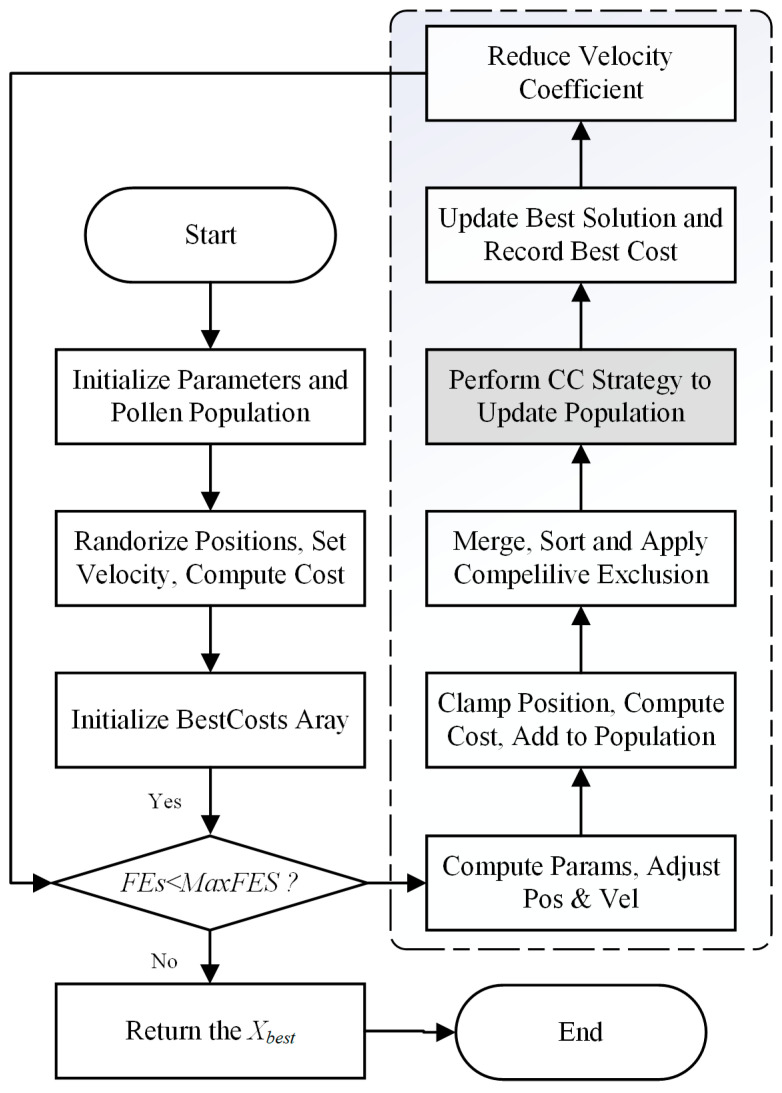
Flowchart of the CCFFO.

After initializing the population, each iteration begins by applying the standard FFO update rules. Subsequently, the CC operator is employed on the resulting population to facilitate multi-dimensional information exchange and generate new trial solutions. A selection mechanism then chooses the fittest individuals from the combined pool to form the next generation. This process repeats until a predefined stopping criterion is met. The complete workflow of CCFFO is depicted in Figure 2.

Algorithm 1 provides the pseudo-code for the CCFFO.
**Algorithm 1** Pseudo-code of the CCFFOSet parameters: MAXFES, population_size N, dim, minL, maxL, β, γ Initialize population P FEs ← 0 **For**
 i = 1:N     P(i).Position ← Random value in [minL, maxL]     P(i).Velocity ← P(i).Position     P(i).Cost ← f(P(i).Position)     FEs ← FEs +1 **End For** Sort population by Cost in ascending order GlobalBest ← P(1) **While** FEs < MAXFES     newPollen ← Initialize empty population     **For** each pollen in P        Calculate K = (X_Best + X_Median + X_Worst)/3        Generate a Lévy step size L        Compute ∆S = L ∗ (X_current − V_current)        Update velocity V_new = V_old ∗ e^(−1/(γt + 1))        Update position X_new = X_current − V_new + ∆S − K ∗ rand()        Clamp X_new to [minL, maxL]        Compute cost of X_new        FEs ← FEs + 1        Add the new pollen to newPollen     **End For**     Merge P and newPollen     Sort the merged population by Cost in ascending order     P ← Retain the top N agents from the sorted population     GlobalBest ← P(1)     /* CC Strategy */     **For** i = 1:N        Perform Horizontal crossover search to update P(i).Position        Perform Vertical crossover search to update P(i).Position        P(i).Cost ← f(P(i).Position)        FEs ← FEs + 1     **End For**     Sort population by Cost in ascending order     GlobalBest ← P(1)     Update γ_new = γ_old ∗ β **End While** **Return** GlobalBest

The computational complexity of CCFFO is determined by four main components: population initialization, fitness evaluation, the standard FFO updates, and the integrated crisscross (CC) operator. Let T be the maximum number of iterations, N be the population size, and D be the problem dimension. The total complexity is therefore the sum of these operations: O(initialization) + O(fitness evaluation) + O(FFO update) + O(CC operator). By identifying the dominant term, the overall computational complexity of CCFFO simplifies to O(T × N × D). Although the CC operator adds computational steps, its complexity remains the same, indicating that it does not increase the overall asymptotic complexity of the algorithm, ensuring that CCFFO maintains the same level of computational efficiency as its predecessor and other common metaheuristic algorithms, such as CSO.

## 4. Experimental Results and Analysis

A rigorous empirical investigation was conducted to assess the optimization capabilities of the proposed CCFFO algorithm. The CEC2017 test suite, comprising 29 benchmark functions, served as the primary testbed for this quantitative assessment. To ensure an unbiased comparison, the parameters for CCFFO were adopted from the original FFO study, and all algorithms were parameterized identically, with a population size of 30 and problem dimensionality of 30. The search process was constrained by a budget of 300,000 function evaluations. To address the stochastic nature of metaheuristics, 30 independent trials were performed for each function, with the mean and standard deviation of the outcomes used as the primary performance metrics.

### 4.1. Benchmark Functions Overview

The performance evaluation is based on the 29 functions from the CEC2017 test suite [29]. The CEC2017 suite encompasses four distinct function categories—unimodal, multimodal, hybrid, and composition—facilitating a thorough evaluation of algorithm performance across diverse fitness landscapes. Details of these benchmark functions are provided in Table 1.

### 4.2. Comparative Analysis on Benchmark Functions

This section presents a comprehensive comparative analysis of the proposed Crisscross Flower Fertilization Optimization (CCFFO) algorithm against its original counterpart, FFO, and eight other widely recognized metaheuristic algorithms: DE [18], GWO [30], MFO [31], SCA [32], PSO [33], PO [34], CSA [35], and HGS [36]. The performance evaluation was conducted on the CEC2017 benchmark suite, a standard for assessing optimization algorithms.

Table 2 provides a detailed summary of the experimental results, reporting the average fitness value (Avg) and standard deviation (Std) obtained by each algorithm over multiple independent runs. Furthermore, to provide a holistic measure of overall performance, the final column of the table presents the overall rank of each algorithm based on the Friedman test across all 29 benchmark functions. In Table 2, the “AVG” column under “Overall Rank” denotes the average ranking calculated from the Friedman test, while the “+/−/=“ column offers the “win/draw/loss” statistics for CCFFO compared to each of the other algorithms.

The results in Table 2 demonstrate the superior performance of the proposed CCFFO. It achieved the top overall rank with an average rank of 1.931, outperforming all competitors. This performance gain is particularly stark when compared to the original FFO, which ranked last with an average of 9.690. This significant improvement highlights the efficacy of the integrated crisscross operator. Moreover, CCFFO surpassed the second-best algorithm, DE (average rank 2.828), by achieving better or equal results on 22 of the 29 functions (17 wins, 5 ties). Finally, the consistently low standard deviations reported for CCFFO across most functions underscore its high stability and reliability.

To further validate these findings, the statistical significance of performance disparities between CCFFO and its counterparts is quantified in Table 3, which reports the *p*-values derived from the Wilcoxon signed-rank test. A *p*-value less than 0.05 is typically considered to indicate a statistically significant difference. The results reveal that CCFFO achieves *p*-values well below 0.05 against the vast majority of algorithms on most functions. For instance, the performance improvement of CCFFO over the original FFO is statistically significant on all 29 functions. This confirms that the improvements are directly attributable to the crisscross strategy, which addresses a key limitation in the original FFO by facilitating structured, multi-dimensional information exchange among individuals. This mechanism enhances population diversity and strengthens the algorithm’s ability to escape from local optima, leading to a more substantial and reliable search performance.

**Figure 3:**
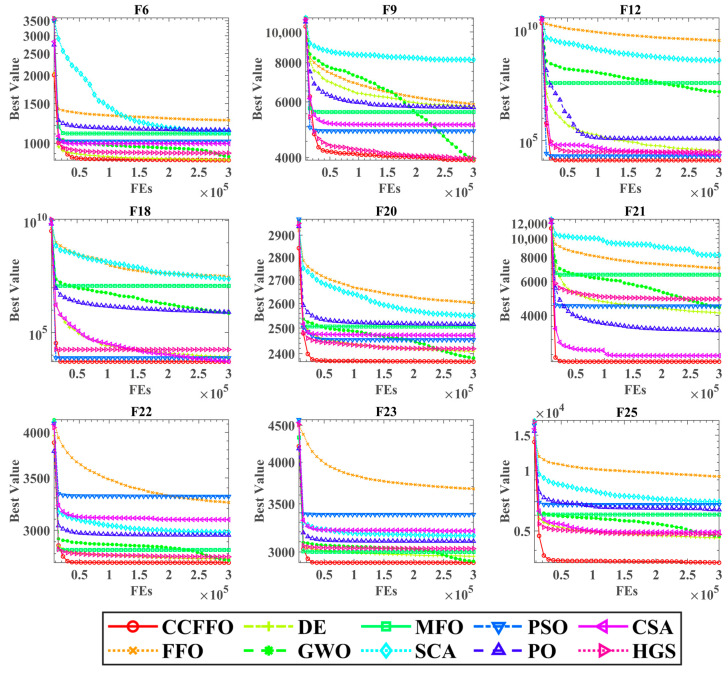
Convergence curves of the CCFFO on benchmarks with other algorithms.

Figure 3 illustrates the convergence profiles of CCFFO and its rival algorithms across various benchmark functions. In these plots, the x-axis denotes the number of function evaluations (FEs), while the y-axis represents the optimal fitness value attained. The plots clearly show that the CCFFO algorithm (solid red line) consistently converges faster and finds better final solutions than all other algorithms. The improvement over the original FFO (dotted orange line) is particularly significant, as FFO often gets stuck at much higher fitness values.

This demonstrates CCFFO’s superior search performance. On functions like F12 and F18, CCFFO shows a rapid initial descent, indicating strong exploitation. On problems like F6 and F20, its steady improvement without stalling highlights effective exploration and the ability to avoid local optima. In short, these curves visually confirm that the crisscross strategy significantly enhances FFO’s optimization capability.

## 5. Application to Production Optimization

The primary objective of the reservoir production application is to determine an optimal operational strategy for a set of production and injection wells that maximizes the field’s economic output, quantified by the Net Present Value (NPV). This task is computationally demanding, as the large number of control variables interacting over a long production lifetime creates a vast and complex search space. This high dimensionality, coupled with the intricate, nonlinear interdependencies between well controls and reservoir response makes metaheuristic algorithms an ideally suited approach for finding robust solutions. In this study, the proposed CCFFO is applied to a three-channel reservoir model, implemented using the MATLAB Reservoir Simulation Toolbox (MRST2024), and its performance is benchmarked against several other leading optimizers. The optimization target is the NPV, which serves as the sole objective function, defined in Equation (6):

(6) $$NPV(x,z)=\sum_{t=1}^{n}\Delta t\cdot \frac{\left(Q_{o,t}\cdot r_{o})-(Q_{w,t}\cdot r_{w})-(Q_{i,t}\cdot r_{i}\right)}{(1+b{)}^{p_{t}}}$$

where x represents the vector of control variables (e.g., well rates), z contains the model’s state parameters, and n is the total number of simulation steps. The terms $Q_{o,t}$, $Q_{w,t}$, and $Q_{i,t}$ signify the production rates of oil and water, and the injection rate of water at time step t, respectively. The parameters $r_{o}$, $r_{w}$, and $r_{i}$ are the per-unit revenue from oil, cost of water disposal, and cost of water injection. Finally, b is the discount rate, and $p_{t}$ is the cumulative time in years corresponding to step t.

### 5.1. Reservoir Model Description

**Figure 4:**
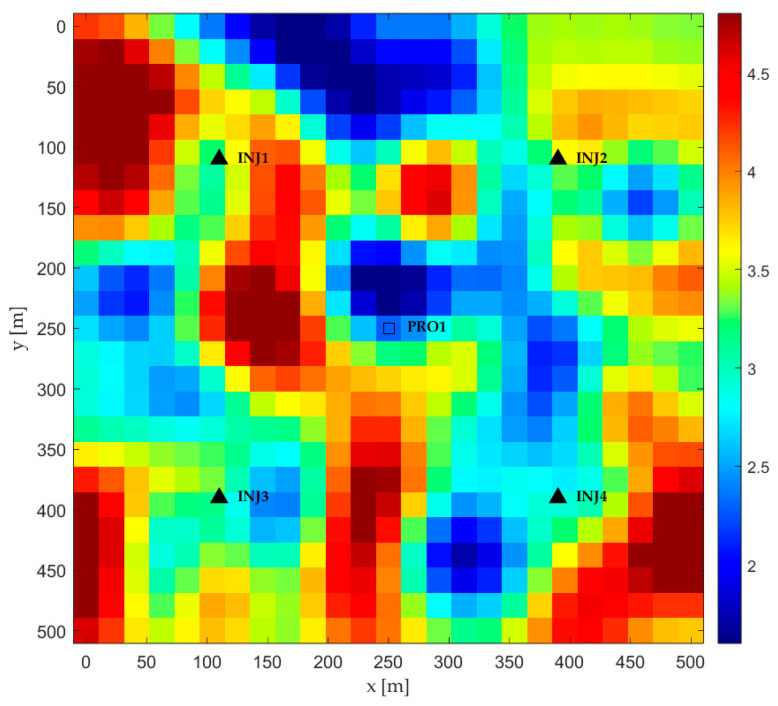
Reservoir Permeability Distribution: Logarithmic Scale.

This research incorporates a case study based on a two-dimensional, synthetic, and heterogeneous reservoir model. It is specifically configured to simulate a complex fluvial channel system. The established layout is a conventional five-spot well pattern, which includes a central production well (PRO1) and four peripheral injection wells (INJ1–INJ4), visually represented in Figure 4. The reservoir space is divided into a 25 × 25 Cartesian grid, comprising 625 active cells. Every grid block measures 20 m by 20 m with consistent thickness. A uniform porosity of 0.2 is also maintained across the entire model.

The heterogeneity of this model stems from its permeability field, which was stochastically generated. This process resulted in clearly delineated high-permeability channels (depicted in red and yellow) and low-permeability barriers (shown in dark blue). Figure 4 provides a visual representation of the spatial distribution of the natural logarithm of permeability, ln(K), highlighting its critical role in dictating fluid flow paths between the injection and production wells. The reservoir begins in a state of oil and water saturation, with the primary objective of the optimization being the efficient management of the waterflooding operation.

The optimization task is defined by the objective to maximize the Net Present Value (NPV). This maximization occurs across a production lifespan totaling 2000 days, segmented into 10 distinct control steps, each lasting 200 days. The set of decision variables encompasses the operational settings for all five wells—specifically, the four injectors and one producer—for every one of these 10 control steps. Consequently, this leads to an optimization problem characterized by a total of 50 dimensions (derived from 5 wells multiplied by 10 control steps).

In this production optimization problem, the optimization variables include the injection rate and the water extraction rate for each well. The injection rate ranges from 0 to 200 STB/DAY, and the water extraction rate for production wells also ranges from 0 to 200 STB/DAY. When all well rates are fixed at 100 STB/DAY, the NPV can be calculated as 7.3287 × 10^7^ USD, which serves as the baseline.

The NPV functions as the fitness criterion for the optimization algorithms, its computation relying on specific economic values. These include an oil price set at 80 USD per stock tank barrel (STB), a water injection expense of 3 USD/STB, and an equivalent water processing charge of 3 USD/STB. To streamline the analysis and concentrate solely on the effectiveness of volumetric recovery, an annual discount rate of 0% is applied within the scope of this study.

### 5.2. Analysis and Discussion of Experimental Results

This section provides a comprehensive performance evaluation of the proposed CCFFO algorithm alongside nine other metaheuristic approaches—FFO, DE, GWO, MFO, SCA, PSO, PO, CSA, and HGS—within the context of the reservoir production optimization problem. Given the computational demands of this application, each algorithm underwent 10 independent executions to ensure a statistically robust comparison. The optimization process was limited to 100 iterations per run. Table 4 compiles the critical statistical indicators, including the average, standard deviation (Std), as well as the highest and lowest Net Present Value (NPV) obtained across these trials.

An examination of the data in Table 4 distinctly highlights CCFFO’s superior efficacy. CCFFO yielded the most favorable average NPV, reaching 9.6172 × 10^7^ USD. This outcome decisively affirms its enhanced capacity to consistently pinpoint highly profitable production schemes. Moreover, CCFFO registered the lowest standard deviation at 1.2971 × 10^6^ USD, a metric that attests to its exceptional operational consistency and dependability when contrasted with all peer algorithms, notably outperforming DE (1.8852 × 10^6^) and HGS (2.0721 × 10^6^).

The performance gain is particularly notable when compared to the original FFO, which not only achieved a significantly lower mean NPV of 8.9711 × 10^7^ USD but also showed nearly double the instability with a standard deviation of 2.1932 × 10^6^. This demonstrates that the crisscross operator enhances both the exploratory power and the exploitation consistency of the algorithm. The quantitative findings underscore CCFFO’s formidable capabilities: adeptly traversing an intricate search landscape to optimize financial gains, concurrent with demonstrating remarkable uniformity in performance across repeated executions.

**Figure 5:**
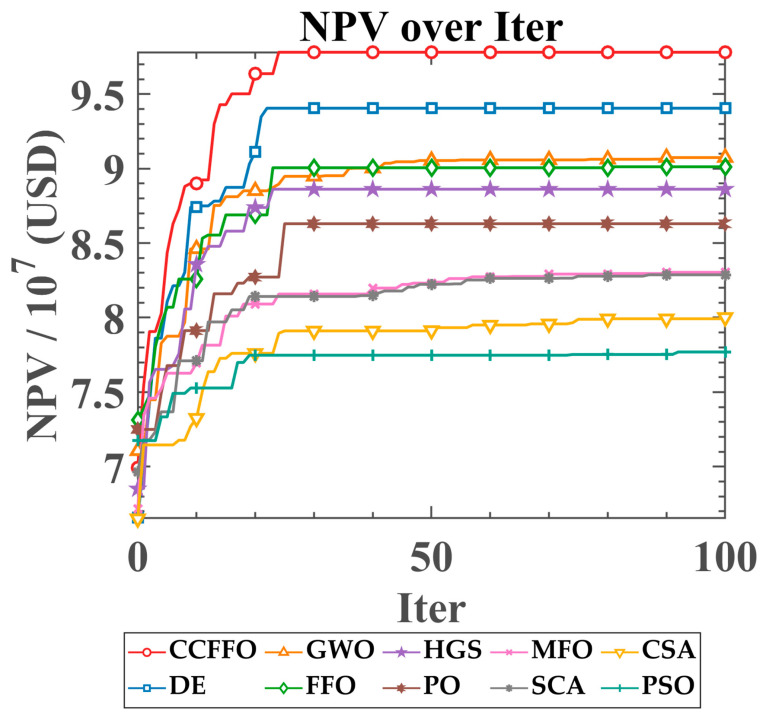
Performance Convergence: NPV Over Iterations.

Figure 5 graphically depicts the Net Present Value (NPV) convergence trajectories for CCFFO and nine comparative algorithms across 100 iterations. With iterations on the horizontal axis and mean NPV on the vertical, CCFFO consistently demonstrates the most rapid convergence and secures the highest ultimate NPV, distinctly surpassing all rival methods.

The algorithm demonstrates a rapid ascent in the initial 30 iterations, quickly identifying a high-value solution and maintaining its superiority throughout the optimization process. In contrast, DE, the second-best algorithm, also converges quickly but stabilizes at a distinctly lower NPV. The performance of the original FFO is significantly inferior to CCFFO, showing both a slower convergence rate and a lower final NPV, which underscores the effectiveness of the integrated crisscross operator. The other algorithms, including GWO and HGS, show moderate performance, while the remaining competitors like PO, MFO, SCA, CSA, and PSO display much slower convergence and ultimately find solutions of considerably lower economic value.

In summary, the convergence plot visually confirms CCFFO’s superior performance. It not only achieves the highest NPV but also converges faster than the other algorithms, demonstrating its efficiency and robustness in solving the complex reservoir optimization problem.

## 6. Conclusions

This study introduced CCFFO, a variant of the Flower Fertilization Optimization algorithm, designed to remedy a structural limitation in its information-sharing mechanism. By integrating a crisscross (CC) operator, CCFFO facilitates direct, multi-dimensional information exchange between individuals. This enhanced communication protocol is designed to generate more diverse and high-quality trial solutions, thereby mitigating premature convergence and improving the algorithm’s overall search efficacy.

The superiority of CCFFO was validated through two comprehensive experiments. First, on the CEC2017 benchmark suite, CCFFO significantly outperformed nine other metaheuristics, achieving the top overall rank and demonstrating a substantial performance gain over the canonical FFO. Second, in its application to a complex reservoir production optimization scenario, CCFFO achieved the maximum NPV and demonstrated the most rapid convergence. This performance substantiates its practical efficacy and resilience when addressing critical engineering problems.

Future research will proceed along two primary avenues. From an algorithmic perspective, efforts will focus on enhancing the scalability of CCFFO for high-dimensional optimization and extending its framework to address multi-objective problems. From an application standpoint, we intend to investigate its performance across a broader spectrum of complex engineering and data science domains to further establish its versatility.

## Figures and Tables

**Table 1:** CEC2017 benchmark functions.

Function	Function Name	Class	Optimum
F1	Shifted and Rotated Bent Cigar Function	Unimodal	100
F2	Shifted and Rotated Zakharov Function	Unimodal	300
F3	Shifted and Rotated Rosenbrock’s Function	Multimodal	400
F4	Shifted and Rotated Rastrigin’s Function	Multimodal	500
F5	Shifted and Rotated Expanded Schaffer’s F6 Function	Multimodal	600
F6	Shifted and Rotated Lunacek Bi-Rastrigin Function	Multimodal	700
F7	Shifted and Rotated Non-Continuous Rastrigin’s Function	Multimodal	800
F8	Shifted and Rotated Lévy Function	Multimodal	900
F9	Shifted and Rotated Schwefel’s Function	Multimodal	1000
F10	Hybrid Function 1 (N = 3)	Hybrid	1100
F11	Hybrid Function 2 (N = 3)	Hybrid	1200
F12	Hybrid Function 3 (N = 3)	Hybrid	1300
F13	Hybrid Function 4 (N = 4)	Hybrid	1400
F14	Hybrid Function 5 (N = 4)	Hybrid	1500
F15	Hybrid Function 6 (N = 4)	Hybrid	1600
F16	Hybrid Function 6 (N = 5)	Hybrid	1700
F17	Hybrid Function 6 (N = 5)	Hybrid	1800
F18	Hybrid Function 6 (N = 5)	Hybrid	1900
F19	Hybrid Function 6 (N = 6)	Hybrid	2000
F20	Composition Function 1 (N = 3)	Composition	2100
F21	Composition Function 2 (N = 3)	Composition	2200
F22	Composition Function 3 (N = 4)	Composition	2300
F23	Composition Function 4 (N = 4)	Composition	2400
F24	Composition Function 5 (N = 5)	Composition	2500
F25	Composition Function 6 (N = 5)	Composition	2600
F26	Composition Function 7 (N = 6)	Composition	2700
F27	Composition Function 8 (N = 6)	Composition	2800
F28	Composition Function 9 (N = 3)	Composition	2900
F29	Composition Function 10 (N = 3)	Composition	3000

**Table 2:** Results of the CCFFO and Other Algorithms on CEC2017.

	F1		F2		F3	
	Avg	Std	Avg	Std	Avg	Std
CCFFO	3.3881 × 10^3^	4.0890 × 10^3^	4.7065 × 10^3^	1.9089 × 10^3^	4.6804 × 10^2^	2.7523 × 10^1^
FFO	3.2720 × 10^10^	9.4736 × 10^9^	7.3720 × 10^4^	6.9991 × 10^3^	7.9199 × 10^3^	4.3161 × 10^3^
DE	1.3949 × 10^3^	2.4372 × 10^3^	1.9102 × 10^4^	4.5464 × 10^3^	4.8701 × 10^2^	1.8843 × 10^0^
GWO	1.5882 × 10^9^	1.2995 × 10^9^	3.4227 × 10^4^	1.2012 × 10^4^	6.3126 × 10^2^	1.8522 × 10^2^
MFO	1.0622 × 10^10^	6.1853 × 10^9^	8.1494 × 10^4^	6.5255 × 10^4^	1.2362 × 10^3^	7.1530 × 10^2^
SCA	1.2621 × 10^10^	1.9108 × 10^9^	3.9515 × 10^4^	5.5319 × 10^3^	1.4592 × 10^3^	2.6218 × 10^2^
PSO	2.5666 × 10^3^	2.7181 × 10^3^	3.0000 × 10^2^	7.6775 × 10^−3^	4.6428 × 10^2^	2.3198 × 10^1^
PO	6.0914 × 10^7^	6.8054 × 10^7^	5.1560 × 10^3^	2.7670 × 10^3^	5.1882 × 10^2^	2.3781 × 10^1^
CSA	2.5045 × 10^3^	3.3705 × 10^3^	3.0001 × 10^2^	8.1843e-03	5.0394 × 10^2^	3.6066 × 10^1^
HGS	8.5018 × 10^3^	7.6934 × 10^3^	1.2486 × 10^3^	2.8687 × 10^3^	4.8301 × 10^2^	3.2918 × 10^1^
	F4		F5		F6	
	Avg	Std	Avg	Std	Avg	Std
CCFFO	6.0979 × 10^2^	2.9360 × 10^1^	6.0000 × 10^2^	9.0260 × 10^−7^	8.4065 × 10^2^	3.9823 × 10^1^
FFO	7.8534 × 10^2^	3.3318 × 10^1^	6.6492 × 10^2^	4.7096 × 10^0^	1.2651 × 10^3^	5.4144 × 10^1^
DE	6.0696 × 10^2^	1.0359 × 10^1^	6.0000 × 10^2^	0.0000 × 10^0^	8.4260 × 10^2^	9.6547 × 10^0^
GWO	6.0753 × 10^2^	2.9419 × 10^1^	6.0657 × 10^2^	3.1605 × 10^0^	8.7307 × 10^2^	4.6072 × 10^1^
MFO	7.1332 × 10^2^	5.7166 × 10^1^	6.3885 × 10^2^	1.2289 × 10^1^	1.1053 × 10^3^	1.7602 × 10^2^
SCA	7.7198 × 10^2^	1.8333 × 10^1^	6.5144 × 10^2^	4.9137 × 10^0^	1.1250 × 10^3^	4.7770 × 10^1^
PSO	7.0426 × 10^2^	3.0113 × 10^1^	6.4486 × 10^2^	9.1094 × 10^0^	1.0207 × 10^3^	6.4600 × 10^1^
PO	7.1488 × 10^2^	4.9183 × 10^1^	6.5833 × 10^2^	9.6541 × 10^0^	1.1449 × 10^3^	8.5649 × 10^1^
CSA	6.9130 × 10^2^	3.2097 × 10^1^	6.4821 × 10^2^	8.4799 × 10^0^	9.9714 × 10^2^	7.1038 × 10^1^
HGS	6.2181 × 10^2^	2.9955 × 10^1^	6.0216 × 10^2^	2.3036 × 10^0^	9.0443 × 10^2^	4.6124 × 10^1^
	F7		F8		F9	
	Avg	Std	Avg	Std	Avg	Std
CCFFO	8.9851 × 10^2^	1.8968 × 10^1^	1.7471 × 10^3^	5.9355 × 10^2^	3.9159 × 10^3^	5.4702 × 10^2^
FFO	1.0194 × 10^3^	2.2381 × 10^1^	6.2413 × 10^3^	3.8207 × 10^2^	5.8887 × 10^3^	6.3741 × 10^2^
DE	9.0837 × 10^2^	1.0189 × 10^1^	9.0000 × 10^2^	9.6743e-14	5.7651 × 10^3^	2.9940 × 10^2^
GWO	8.8904 × 10^2^	2.8199 × 10^1^	1.8415 × 10^3^	5.0349 × 10^2^	3.9461 × 10^3^	4.7637 × 10^2^
MFO	1.0126 × 10^3^	5.1630 × 10^1^	7.7088 × 10^3^	3.6124 × 10^3^	5.5687 × 10^3^	8.1903 × 10^2^
SCA	1.0507 × 10^3^	1.7524 × 10^1^	5.4695 × 10^3^	9.7933 × 10^2^	8.1652 × 10^3^	2.6112 × 10^2^
PSO	9.5080 × 10^2^	3.2770 × 10^1^	4.2856 × 10^3^	6.2042 × 10^2^	4.8491 × 10^3^	5.6152 × 10^2^
PO	9.7420 × 10^2^	3.0895 × 10^1^	4.8893 × 10^3^	8.3169 × 10^2^	5.7592 × 10^3^	7.7801 × 10^2^
CSA	9.3647 × 10^2^	1.6700 × 10^1^	3.5509 × 10^3^	7.7713 × 10^2^	5.0778 × 10^3^	6.1294 × 10^2^
HGS	9.1477 × 10^2^	2.6506 × 10^1^	3.5684 × 10^3^	1.0017 × 10^3^	3.9738 × 10^3^	4.3318 × 10^2^
	F10		F11		F12	
	Avg	Std	Avg	Std	Avg	Std
CCFFO	1.1386 × 10^3^	2.3581 × 10^1^	2.6791 × 10^5^	1.4828 × 10^5^	1.3036 × 10^4^	7.3841 × 10^3^
FFO	4.3646 × 10^3^	1.5122 × 10^3^	4.8593 × 10^9^	2.6705 × 10^9^	3.1396 × 10^9^	3.2213 × 10^9^
DE	1.1643 × 10^3^	2.3151 × 10^1^	1.5039 × 10^6^	6.3398 × 10^5^	3.0019 × 10^4^	1.7935 × 10^4^
GWO	1.8942 × 10^3^	7.9974 × 10^2^	7.6671 × 10^7^	1.0500 × 10^8^	1.5481 × 10^7^	5.5450 × 10^7^
MFO	4.1124 × 10^3^	3.5171 × 10^3^	2.9710 × 10^8^	6.8070 × 10^8^	3.8744 × 10^7^	1.9323 × 10^8^
SCA	2.0866 × 10^3^	2.7922 × 10^2^	1.1559 × 10^9^	2.5813 × 10^8^	4.0446 × 10^8^	1.7268 × 10^8^
PSO	1.2121 × 10^3^	3.1334 × 10^1^	4.1419 × 10^4^	1.9971 × 10^4^	2.0171 × 10^4^	1.8112 × 10^4^
PO	1.3175 × 10^3^	6.6615 × 10^1^	2.0095 × 10^7^	2.4767 × 10^7^	1.2096 × 10^5^	6.0060 × 10^4^
CSA	1.2498 × 10^3^	4.9018 × 10^1^	2.0200 × 10^6^	1.5415 × 10^6^	2.3269 × 10^4^	1.2670 × 10^4^
HGS	1.2216 × 10^3^	3.7360 × 10^1^	9.1223 × 10^5^	7.7382 × 10^5^	3.0920 × 10^4^	2.5837 × 10^4^
	F13		F14		F15	
	Avg	Std	Avg	Std	Avg	Std
CCFFO	2.5111 × 10^4^	2.7365 × 10^4^	2.2860 × 10^3^	1.1378 × 10^3^	2.5904 × 10^3^	2.9099 × 10^2^
FFO	1.4639 × 10^6^	9.7417 × 10^5^	1.1528 × 10^8^	2.1154 × 10^8^	3.7903 × 10^3^	4.2718 × 10^2^
DE	5.1970 × 10^4^	3.9254 × 10^4^	6.9884 × 10^3^	3.3943 × 10^3^	2.0457 × 10^3^	1.4870 × 10^2^
GWO	2.6527 × 10^5^	3.5817 × 10^5^	5.7499 × 10^5^	1.0704 × 10^6^	2.3967 × 10^3^	2.6304 × 10^2^
MFO	2.0365 × 10^5^	4.2357 × 10^5^	8.6397 × 10^4^	1.2295 × 10^5^	3.0842 × 10^3^	3.8883 × 10^2^
SCA	1.2206 × 10^5^	6.5504 × 10^4^	1.0715 × 10^7^	1.0103 × 10^7^	3.5441 × 10^3^	2.5708 × 10^2^
PSO	7.9298 × 10^3^	6.2803 × 10^3^	8.7293 × 10^3^	9.5830 × 10^3^	2.9752 × 10^3^	3.3283 × 10^2^
PO	4.5665 × 10^4^	2.8233 × 10^4^	6.1031 × 10^4^	6.3175 × 10^4^	3.2313 × 10^3^	4.0381 × 10^2^
CSA	1.6431 × 10^3^	6.1823 × 10^1^	1.0536 × 10^4^	5.9958 × 10^3^	2.9307 × 10^3^	3.1545 × 10^2^
HGS	4.0731 × 10^4^	3.3024 × 10^4^	1.9925 × 10^4^	1.5766 × 10^4^	2.6777 × 10^3^	2.9716 × 10^2^
	F16		F17		F18	
	Avg	Std	Avg	Std	Avg	Std
CCFFO	2.0061 × 10^3^	1.6557 × 10^2^	2.5342 × 10^5^	1.9617 × 10^5^	5.1152 × 10^3^	1.6276 × 10^3^
FFO	2.7891 × 10^3^	6.1977 × 10^2^	1.2063 × 10^7^	2.4887 × 10^7^	3.0165 × 10^7^	6.5274 × 10^7^
DE	1.8430 × 10^3^	4.1259 × 10^1^	3.3449 × 10^5^	1.7845 × 10^5^	8.2305 × 10^3^	4.5262 × 10^3^
GWO	2.0128 × 10^3^	1.6275 × 10^2^	5.9358 × 10^5^	1.0739 × 10^6^	7.2434 × 10^5^	1.6454 × 10^6^
MFO	2.5180 × 10^3^	2.3231 × 10^2^	3.6811 × 10^6^	7.2508 × 10^6^	1.2124 × 10^7^	3.6755 × 10^7^
SCA	2.3980 × 10^3^	1.8503 × 10^2^	2.8225 × 10^6^	1.7072 × 10^6^	2.4757 × 10^7^	1.1562 × 10^7^
PSO	2.3648 × 10^3^	2.3968 × 10^2^	1.5927 × 10^5^	1.0753 × 10^5^	7.4746 × 10^3^	9.8525 × 10^3^
PO	2.3028 × 10^3^	2.4955 × 10^2^	5.5040 × 10^5^	4.3576 × 10^5^	8.2365 × 10^5^	6.6923 × 10^5^
CSA	2.2590 × 10^3^	2.3853 × 10^2^	2.4300 × 10^4^	1.1406 × 10^4^	5.2186 × 10^3^	5.8926 × 10^3^
HGS	2.3202 × 10^3^	1.9175 × 10^2^	2.9100 × 10^5^	2.5191 × 10^5^	1.7732 × 10^4^	1.6537 × 10^4^
	F19		F20		F21	
	Avg	Std	Avg	Std	Avg	Std
CCFFO	2.2693 × 10^3^	1.3807 × 10^2^	2.3694 × 10^3^	3.6153 × 10^1^	2.3000 × 10^3^	3.5827e-13
FFO	2.5874 × 10^3^	1.3039 × 10^2^	2.6041 × 10^3^	4.8142 × 10^1^	7.0276 × 10^3^	1.0755 × 10^3^
DE	2.1296 × 10^3^	8.1035 × 10^1^	2.4093 × 10^3^	1.0166 × 10^1^	4.1104 × 10^3^	2.0812 × 10^3^
GWO	2.3629 × 10^3^	1.4547 × 10^2^	2.3832 × 10^3^	1.6838 × 10^1^	4.3958 × 10^3^	1.6311 × 10^3^
MFO	2.7506 × 10^3^	1.9564 × 10^2^	2.5059 × 10^3^	3.8579 × 10^1^	6.4952 × 10^3^	1.5493 × 10^3^
SCA	2.5935 × 10^3^	1.1895 × 10^2^	2.5506 × 10^3^	1.8541 × 10^1^	8.2068 × 10^3^	2.4221 × 10^3^
PSO	2.6340 × 10^3^	2.2488 × 10^2^	2.4535 × 10^3^	5.6795 × 10^1^	4.4673 × 10^3^	2.2543 × 10^3^
PO	2.5393 × 10^3^	1.7828 × 10^2^	2.5153 × 10^3^	5.4875 × 10^1^	3.3303 × 10^3^	1.8239 × 10^3^
CSA	2.4747 × 10^3^	1.2842 × 10^2^	2.4713 × 10^3^	4.1592 × 10^1^	2.4707 × 10^3^	9.2913 × 10^2^
HGS	2.4980 × 10^3^	2.0102 × 10^2^	2.4187 × 10^3^	2.8084 × 10^1^	4.8602 × 10^3^	1.5246 × 10^3^
	F22		F23		F24	
	Avg	Std	Avg	Std	Avg	Std
CCFFO	2.7272 × 10^3^	2.2919 × 10^1^	2.8987 × 10^3^	1.8880 × 10^1^	2.8939 × 10^3^	1.5141 × 10^1^
FFO	3.2560 × 10^3^	1.4457 × 10^2^	3.6733 × 10^3^	2.8431 × 10^2^	3.7793 × 10^3^	4.3463 × 10^2^
DE	2.7578 × 10^3^	7.9885 × 10^0^	2.9590 × 10^3^	1.2101 × 10^1^	2.8874 × 10^3^	3.9882 × 10^−1^
GWO	2.7478 × 10^3^	3.3344 × 10^1^	2.9121 × 10^3^	3.3407 × 10^1^	2.9764 × 10^3^	3.5718 × 10^1^
MFO	2.8316 × 10^3^	3.5175 × 10^1^	3.0006 × 10^3^	4.2546 × 10^1^	3.2953 × 10^3^	4.8754 × 10^2^
SCA	2.9844 × 10^3^	2.6955 × 10^1^	3.1613 × 10^3^	2.3055 × 10^1^	3.1830 × 10^3^	5.7385 × 10^1^
PSO	3.3144 × 10^3^	1.7054 × 10^2^	3.3819 × 10^3^	1.0675 × 10^2^	2.8817 × 10^3^	1.2739 × 10^1^
PO	2.9581 × 10^3^	7.0920 × 10^1^	3.1055 × 10^3^	6.3218 × 10^1^	2.9414 × 10^3^	2.6784 × 10^1^
CSA	3.0959 × 10^3^	1.0424 × 10^2^	3.2092 × 10^3^	1.2090 × 10^2^	2.9316 × 10^3^	2.1021 × 10^1^
HGS	2.7752 × 10^3^	3.1514 × 10^1^	3.0324 × 10^3^	4.8507 × 10^1^	2.8883 × 10^3^	7.5245 × 10^0^
	F25		F26		F27	
	Avg	Std	Avg	Std	Avg	Std
CCFFO	3.4728 × 10^3^	1.1033 × 10^3^	3.2210 × 10^3^	1.0616 × 10^1^	3.1424 × 10^3^	4.7079 × 10^1^
FFO	9.3492 × 10^3^	7.9015 × 10^2^	3.8081 × 10^3^	2.2961 × 10^2^	5.2444 × 10^3^	5.5411 × 10^2^
DE	4.6204 × 10^3^	1.0661 × 10^2^	3.2060 × 10^3^	2.9832 × 10^0^	3.1768 × 10^3^	5.6392 × 10^1^
GWO	4.7008 × 10^3^	3.8852 × 10^2^	3.2534 × 10^3^	2.9753 × 10^1^	3.4148 × 10^3^	9.1294 × 10^1^
MFO	6.0385 × 10^3^	4.9203 × 10^2^	3.2464 × 10^3^	2.0168 × 10^1^	4.5376 × 10^3^	9.7285 × 10^2^
SCA	7.0123 × 10^3^	2.9692 × 10^2^	3.4056 × 10^3^	3.4502 × 10^1^	3.8128 × 10^3^	1.2535 × 10^2^
PSO	6.7644 × 10^3^	2.1414 × 10^3^	3.2406 × 10^3^	2.7363 × 10^2^	3.1621 × 10^3^	6.3606 × 10^1^
PO	6.3874 × 10^3^	1.4138 × 10^3^	3.3174 × 10^3^	7.8114 × 10^1^	3.2975 × 10^3^	3.5404 × 10^1^
CSA	4.9366 × 10^3^	2.2006 × 10^3^	3.6000 × 10^3^	1.8595 × 10^2^	3.2145 × 10^3^	1.8546 × 10^1^
HGS	4.8055 × 10^3^	4.8203 × 10^2^	3.2264 × 10^3^	1.4534 × 10^1^	3.1937 × 10^3^	5.3486 × 10^1^
	F28		F29			
	Avg	Std	Avg	Std		
CCFFO	3.5697 × 10^3^	1.7701 × 10^2^	6.5019 × 10^3^	7.9346 × 10^2^		
FFO	5.3737 × 10^3^	6.4659 × 10^2^	1.4443 × 10^8^	5.4055 × 10^8^		
DE	3.5174 × 10^3^	6.2973 × 10^1^	1.1730 × 10^4^	2.0397 × 10^3^		
GWO	3.7025 × 10^3^	1.2777 × 10^2^	5.0522 × 10^6^	4.5566 × 10^6^		
MFO	4.2545 × 10^3^	3.4156 × 10^2^	4.7871 × 10^5^	5.8888 × 10^5^		
SCA	4.6363 × 10^3^	2.3954 × 10^2^	7.2163 × 10^7^	3.8671 × 10^7^		
PSO	4.0002 × 10^3^	2.8736 × 10^2^	5.3397 × 10^3^	2.7270 × 10^3^		
PO	4.5231 × 10^3^	3.4208 × 10^2^	7.1810 × 10^6^	5.2589 × 10^6^		
CSA	4.4017 × 10^3^	3.6065 × 10^2^	1.1669 × 10^5^	9.3591 × 10^4^		
HGS	3.7414 × 10^3^	1.9507 × 10^2^	1.2730 × 10^5^	1.6515 × 10^5^		
	**Overall Rank**					
	**RANK**	+/=/−	**AVG**			
CCFFO	1	~	1.931			
FFO	10	29/0/0	9.6897			
DE	2	17/5/7	2.8276			
GWO	6	21/7/1	4.7931			
MFO	8	29/0/0	7.4828			
SCA	9	29/0/0	8.5172			
PSO	4	18/5/6	4.4138			
PO	7	28/1/0	6.5517			
CSA	5	25/1/3	4.5862			
HGS	3	21/7/1	4.2069			

**Table 3:** The *p*-values of the CCFFO versus other algorithms on CEC2017.

	CCFFO	FFO	DE	GWO	MFO
F1	/	1.7344 × 10^−6^	8.7297 × 10^−3^	1.7344 × 10^−6^	1.7344 × 10^−6^
F2	/	1.7344 × 10^−6^	1.7344 × 10^−6^	1.7344 × 10^−6^	6.9838 × 10^−6^
F3	/	1.7344 × 10^−6^	1.3820 × 10^−3^	1.7344 × 10^−6^	1.7344 × 10^−6^
F4	/	1.7344 × 10^−6^	9.7539 × 10^−1^	4.5281 × 10^−1^	2.8786 × 10^−6^
F5	/	1.7344 × 10^−6^	3.9063 × 10^−3^	1.7344 × 10^−6^	1.7344 × 10^−6^
F6	/	1.7344 × 10^−6^	7.0356 × 10^−1^	1.1079 × 10^−2^	2.1266 × 10^−6^
F7	/	1.7344 × 10^−6^	1.8519 × 10^−2^	7.1903 × 10^−2^	1.7344 × 10^−6^
F8	/	1.7344 × 10^−6^	1.7344 × 10^−6^	1.8462 × 10^−1^	1.7344 × 10^−6^
F9	/	2.3534 × 10^−6^	1.7344 × 10^−6^	8.6121 × 10^−1^	2.8786 × 10^−6^
F10	/	1.7344 × 10^−6^	3.8811 × 10^−4^	1.7344 × 10^−6^	1.7344 × 10^−6^
F11	/	1.7344 × 10^−6^	2.1266 × 10^−6^	1.7344 × 10^−6^	2.8786 × 10^−6^
F12	/	1.7344 × 10^−6^	4.8603 × 10^−5^	1.7344 × 10^−6^	1.7344 × 10^−6^
F13	/	1.7344 × 10^−6^	1.1973 × 10^−3^	1.7423 × 10^−4^	1.3820 × 10^−3^
F14	/	1.7344 × 10^−6^	2.8786 × 10^−6^	1.7344 × 10^−6^	1.7344 × 10^−6^
F15	/	1.7344 × 10^−6^	2.8786 × 10^−6^	3.3789 × 10^−3^	1.2506 × 10^−4^
F16	/	1.9209 × 10^−6^	1.3595 × 10^−4^	9.0993 × 10^−1^	2.8786 × 10^−6^
F17	/	1.7344 × 10^−6^	1.2044 × 10^−1^	2.4308 × 10^−2^	4.5336 × 10^−4^
F18	/	1.7344 × 10^−6^	2.2551 × 10^−3^	1.7344 × 10^−6^	6.9838 × 10^−6^
F19	/	1.1265 × 10^−5^	1.0357 × 10^−3^	1.3975 × 10^−2^	2.3534 × 10^−6^
F20	/	1.7344 × 10^−6^	2.1266 × 10^−6^	8.2206 × 10^−2^	1.7344 × 10^−6^
F21	/	1.7344 × 10^−6^	1.7344 × 10^−6^	1.7344 × 10^−6^	1.7344 × 10^−6^
F22	/	1.7344 × 10^−6^	5.2165 × 10^−6^	4.7162 × 10^−2^	1.7344 × 10^−6^
F23	/	1.7344 × 10^−6^	1.7344 × 10^−6^	1.5286 × 10^−1^	1.7344 × 10^−6^
F24	/	1.7344 × 10^−6^	3.4935 × 10^−1^	1.7344 × 10^−6^	1.7344 × 10^−6^
F25	/	1.7344 × 10^−6^	1.0570 × 10^−4^	1.1499 × 10^−4^	2.8786 × 10^−6^
F26	/	1.7344 × 10^−6^	2.6033 × 10^−6^	4.2857 × 10^−6^	3.1123 × 10^−5^
F27	/	1.7344 × 10^−6^	1.3194 × 10^−2^	1.7344 × 10^−6^	1.7344 × 10^−6^
F28	/	1.7344 × 10^−6^	2.0589 × 10^−1^	1.4839 × 10^−3^	2.3534 × 10^−6^
F29	/	1.7344 × 10^−6^	1.7344 × 10^−6^	1.7344 × 10^−6^	1.7344 × 10^−6^
	SCA	PSO	PO	CSA	HGS
F1	1.7344 × 10^−6^	7.3433 × 10^−1^	1.7344 × 10^−6^	1.9152 × 10^−1^	1.2866 × 10^−3^
F2	1.7344 × 10^−6^	1.7344 × 10^−6^	5.4401 × 10^−1^	1.7344 × 10^−6^	1.3595 × 10^−4^
F3	1.7344 × 10^−6^	9.7539 × 10^−1^	5.2165 × 10^−6^	2.6134 × 10^−4^	6.2683 × 10^−2^
F4	1.7344 × 10^−6^	1.7344 × 10^−6^	1.7344 × 10^−6^	2.6033 × 10^−6^	8.9718 × 10^−2^
F5	1.7344 × 10^−6^	1.7344 × 10^−6^	1.7344 × 10^−6^	1.7344 × 10^−6^	1.7344 × 10^−6^
F6	1.7344 × 10^−6^	1.7344 × 10^−6^	1.7344 × 10^−6^	1.9209 × 10^−6^	1.7988 × 10^−5^
F7	1.7344 × 10^−6^	1.0246 × 10^−5^	1.7344 × 10^−6^	3.5152 × 10^−6^	3.8723 × 10^−2^
F8	1.7344 × 10^−6^	1.9209 × 10^−6^	1.7344 × 10^−6^	1.9209 × 10^−6^	4.7292 × 10^−6^
F9	1.7344 × 10^−6^	8.9187 × 10^−5^	1.9209 × 10^−6^	8.4661 × 10^−6^	7.4987 × 10^−1^
F10	1.7344 × 10^−6^	4.2857 × 10^−6^	1.7344 × 10^−6^	1.7344 × 10^−6^	2.6033 × 10^−6^
F11	1.7344 × 10^−6^	2.3534 × 10^−6^	1.7344 × 10^−6^	2.1266 × 10^−6^	1.0357 × 10^−3^
F12	1.7344 × 10^−6^	1.4139 × 10^−1^	1.7344 × 10^−6^	1.8326 × 10^−3^	1.1138 × 10^−3^
F13	2.6033 × 10^−6^	8.9443 × 10^−4^	6.4242 × 10^−3^	1.7344 × 10^−6^	4.9498 × 10^−2^
F14	1.7344 × 10^−6^	3.0650 × 10^−4^	1.7344 × 10^−6^	1.7344 × 10^−6^	2.8786 × 10^−6^
F15	2.1266 × 10^−6^	1.2506 × 10^−4^	1.2381 × 10^−5^	8.9443 × 10^−4^	2.0589 × 10^−1^
F16	2.1266 × 10^−6^	2.8434 × 10^−5^	4.8603 × 10^−5^	2.5967 × 10^−5^	4.7292 × 10^−6^
F17	1.7344 × 10^−6^	3.8723 × 10^−2^	1.1138 × 10^−3^	1.7344 × 10^−6^	6.4352 × 10^−1^
F18	1.7344 × 10^−6^	5.3044 × 10^−1^	1.7344 × 10^−6^	2.3038 × 10^−2^	1.1499 × 10^−4^
F19	6.3391 × 10^−6^	4.2857 × 10^−6^	4.2857 × 10^−6^	1.9729 × 10^−5^	6.3198 × 10^−5^
F20	1.7344 × 10^−6^	3.1123 × 10^−5^	1.7344 × 10^−6^	1.7344 × 10^−6^	5.7517 × 10^−6^
F21	1.7344 × 10^−6^	1.3101 × 10^−4^	1.7344 × 10^−6^	1.7344 × 10^−6^	1.7344 × 10^−6^
F22	1.7344 × 10^−6^	1.7344 × 10^−6^	1.7344 × 10^−6^	1.7344 × 10^−6^	5.2165 × 10^−6^
F23	1.7344 × 10^−6^	1.7344 × 10^−6^	1.7344 × 10^−6^	1.7344 × 10^−6^	1.7344 × 10^−6^
F24	1.7344 × 10^−6^	5.2872 × 10^−4^	5.2165 × 10^−6^	2.5967 × 10^−5^	8.9718 × 10^−2^
F25	1.7344 × 10^−6^	2.5970 × 10^−5^	3.5152 × 10^−6^	1.8326 × 10^−3^	2.0515 × 10^−4^
F26	1.7344 × 10^−6^	3.5888 × 10^−4^	1.7344 × 10^−6^	1.7344 × 10^−6^	8.2206 × 10^−2^
F27	1.7344 × 10^−6^	2.7116 × 10^−1^	1.7344 × 10^−6^	3.1817 × 10^−6^	2.8308 × 10^−4^
F28	1.7344 × 10^−6^	9.3157 × 10^−6^	1.7344 × 10^−6^	1.9209 × 10^−6^	2.9575 × 10^−3^
F29	1.7344 × 10^−6^	7.2710 × 10^−3^	1.7344 × 10^−6^	1.7344 × 10^−6^	1.7344 × 10^−6^

**Table 4:** The results of CCFFO and other algorithms on the oil reservoir production optimization.

Algorithm	NPV (USD)			
	Mean	Std	Best	Worst
CCFFO	9.6172 × 10^7^	1.2971 × 10^6^	9.9131 × 10^7^	9.3212 × 10^7^
FFO	8.9711 × 10^7^	2.1932 × 10^6^	9.3259 × 10^7^	8.4846 × 10^7^
DE	9.2935 × 10^7^	1.8852 × 10^6^	9.7418 × 10^7^	8.8915 × 10^7^
GWO	9.0331 × 10^7^	2.2142 × 10^6^	9.5422 × 10^7^	8.5497 × 10^7^
MFO	8.2722 × 10^7^	1.9501 × 10^6^	8.6192 × 10^7^	7.7980 × 10^7^
SCA	8.2555 × 10^7^	2.9418 × 10^6^	8.7861 × 10^7^	7.6303 × 10^7^
PSO	7.7534 × 10^7^	3.6438 × 10^6^	8.6419 × 10^7^	7.0297 × 10^7^
PO	8.5447 × 10^7^	2.4544 × 10^6^	8.9692 × 10^7^	7.9498 × 10^7^
CSA	7.9733 × 10^7^	3.5607 × 10^6^	8.5280 × 10^7^	7.1727 × 10^7^
HGS	8.8127 × 10^7^	2.0721 × 10^6^	9.2660 × 10^7^	8.3578 × 10^7^

## Data Availability

The numerical and experimental data used to support the findings of this study are included within the article.
